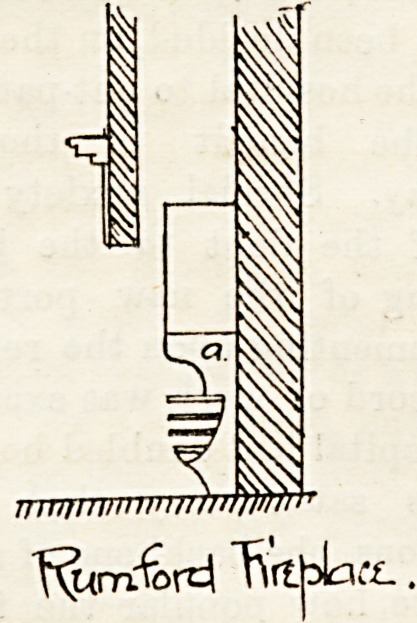# Heating and Ventilation

**Published:** 1895-03-09

**Authors:** 


### PRACTICAL DEPARTMENTS.

### HEATING AND VENTILATION.

Fire-places Ancient and Modern.

The gradual changes which have taken place in the form and construction of fire-places, and generally in the various systems of heating and ventilation of private houses and public institutions, are worthy of study. The history of the fire-place, traced back from its beginnings down to its present developments, is interesting in many respects. It is not until these developments are so traced that it is possible to realise how slowly improvements have been effected, and how far we still are from having arrived at anything like a universal understanding of the first principles to be applied in making our houses and public buildings healthy, warm, and comfortable.

Questions of warming and ventilating are so naturally and closely connected as to be practically inseparable, although the connection is one which is not "understanded" of the jerry builder or the amateur architect, as is abundantly and painfully testified by the smoky chimneys and draughty rooms of modern villas, those apologies for "houses" which are found springing up everywhere, put together in a few weeks, and which are as highly rented as they are badly built.

In a book published some years ago by Frederick Edwards, jun., on "Our Domestic Fire-places," there are to be found some interesting facts in the history of warming apparatus. It is curious that although the Romans, to go back to very early times, had no knowledge of the use of chimneys, yet they were in the habit of heating their houses by a more or less elaborate system of hot-air flues beneath the floors. Through the action of a fire kindled beneath the floor at one end of a building, the hot air and smoke escaped through chambers to an opening at the opposite side. The Greeks, instead of "concerning themselves with devising methods for discharging smoke into the open air," "confined their attention to the apparently more useful object of avoiding the formation of smoke and any other unpleasant effects of burning fuel." "Fuel" at this time, of course, consisted principally of wood, generally green. Smoke must have been the normal atmosphere of the living rooms of dwellings in cold weather. A relic of these primitive customs is found in the open "brazier" still much used in some European countries, notably, according to Mr. Edwards, in Italy and Spain; and in England, even as late as the end of the last century, "large areas, including the chamber of the House of Commons, were heated by charcoal or coke burned in the open brazier."

Chimneys were apparently not made use of in England till the twelfth or thirteenth century. At Connisborough Castle, in Yorkshire, is to be seen a specimen of the earliest effort in that direction, a first departure from the holes in floor and roof which constituted the open fire-place in Norman and Saxon times. Here the fire was placed against an external wall instead of occupying the centre of the room, and the smoke escaped by means of a slanting aperture behind. Then by degrees the chimney as we know it crept into use. First it was introduced into the principal rooms of dwellings, and then by building one chimney in front of another in more than one storey. It was not until the seventeenth century that these were built side by side as at present, so as to avoid the projections occasioned in the upper rooms.

Section of Prince Ruperts Fireplace.

Rumford Fireplace.

Coal as fuel was very gradually introduced. In 1306 Parliament "petitioned the King to prohibit the use of the noxious fuel within the City, it being the opinion of the citizens that the fumes corrupted the air and were injurious to health." In the reign of Edward I. a man was "tried, convicted, and executed for the crime of burning sea coal in London."

The first equivalent to a modern grate consisted of an iron "cradle," necessitated by the gradual introduction of coal as fuel, made probably of a few "bars which were bent, joined together, and attached to the back of the fire-place somewhat after the fashion of a horse-rack in a stable." Now by degrees, with the adoption of coal for domestic purposes, the huge open chimneys were replaced by more contracted openings, so made with a view to lessen the smoke. "In 1594 Sir Hugh Platt, a lawyer, recommended the contraction of the fire-place by a false brick back and sides, on the warranty of a gentleman from Ireland, who was 'a great practiser of artificial conclusions'"; and in 1658 Sir John Winter, a cousin of the Marquis of Worcester, invented a plan for increasing the combustion of the coal and hindering smoke by introducing below the fire-cage an iron box, communicating with the external air by means of a tube. In the illustrations here reproduced from Mr. Edwards' book, the first shows a section of a fire-place invented by Prince Rupert, who applies to it a principle much in use now, but which was almost entirely neglected from the time of its author till within the memory of the present generation. It is that of "allowing the smoke to pass away by a narrow aperture behind the grate instead of rising direct to the chimney from the fire." To lessen the draught of the fire a door can be pulled forward (*a*) to compel the smoke to descend before entering the chimney, and there is also provided a blower (*b*) to increase the draught if desired.

Coming down to more modern times, great improvements were effected by Count Rumford at the end of the last and beginning of the present century, who introduced a reduction in the size of the fire-place, the use of brickwork instead of iron, and splaying the brick sides to increase radiation of heat. Sir Douglas Galton, in his recent book on "Hospital Construction," says, "So far as radiant heat alone is concerned, it is difficult to improve upon the simple form of 'Rumford grate,' with splayed firebrick sides, and with the back arranged to lean slightly forwards over the fire, whilst in order to favour the draught some air should be admitted through the bottom of the grate, and the front bars should be vertical to prevent accumulation of ashes upon them." The principle is shown in the second illustration. There is a domed surface of firebrick (*a*) over the fire, and a contracted entrance to the chimney.

(*To be continued.*)

## Figures and Tables

**Figure f1:**
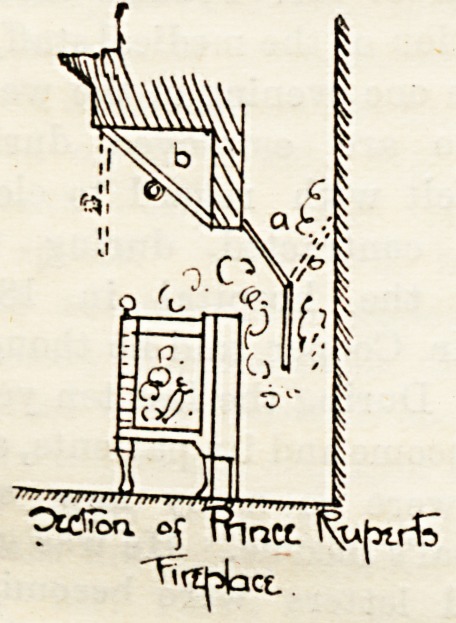


**Figure f2:**